# Sagittal Knee Gait Changes After Medial Unicompartmental and Total Knee Arthroplasty: An Exploratory Analysis of 32 Patients

**DOI:** 10.7759/cureus.73188

**Published:** 2024-11-07

**Authors:** Julius T Hald, Jacob F Mortensen, Emil Gleipner-Andersen, Leah Lehmann, Asger M Haugaard, Thomas Scheike, Anders Odgaard

**Affiliations:** 1 Orthopaedic Surgery, Rigshospitalet, Copenhagen, DNK; 2 Orthopaedic Surgery, Zealand University Hospital, Nykøbing Falster, DNK; 3 Orthopaedic Surgery, Gentofte Hospital, Copenhagen, DNK; 4 Orthopaedic Surgery, Zealand University Hospital, Køge, DNK; 5 Biostatistics, Copenhagen University, Copenhagen, DNK

**Keywords:** biomechanics, gait analysis, knee arthroplasty, medial unicompartmental knee arthroplasty, total knee arthroplasty

## Abstract

Background: This study aimed to investigate postoperative developments of sagittal knee gait in a population of knee arthroplasty patients randomized to either unicompartmental or total knee arthroplasty. We hypothesized that knee arthroplasty patients develop greater walking speeds, range of motion, sagittal knee angle velocities, and sagittal knee angle accelerations.

Methods: Thirty-two patients were recruited from a randomized trial comparing the two implant types. Sagittal knee gait was examined preoperatively and at four and 12 months postoperatively. The examination used inertial measurement units. Nine gait parameters were defined focusing on knee angles, angular velocities, and accelerations.

Results: Stride frequency increased by 0.2 s^-1^. Walking speed increased by 0.3 m/s. The range of motion increased by 7 degrees. Extension and flexion velocity during knee swing increased by 72 and 49 degrees/second. Acceleration during flexion increased by 565 degrees/second^2^. Acceleration during extension increased by 1168 degrees/second^2^. Acceleration after heel strike increased by 1549 degrees/second^2^.

Conclusion: We observed significant developments in sagittal knee gait after knee arthroplasty. Patients developed faster walking speed and greater stride frequency, as well as improvements in the range of motion, sagittal knee angle velocities, and accelerations.

## Introduction

Knee arthroplasty is a safe and effective treatment option for idiopathic knee osteoarthritis [1]. The incidence of patients suffering from knee osteoarthritis has been increasing rapidly over the last 20 years, resulting in increasing rates of knee arthroplasty [1]. A study has projected a 600 percent increase in knee arthroplasty rates from 450,000 procedures in 2005 to 3.48 million procedures by 2030 in the United States [2]. The decision of performing the procedure is made jointly by the patient and the knee surgeon depending on patient's symptoms and radiographic evidence of arthritis [1]. No clear consensus exists regarding the exact indications as they are not completely objective but based on the patient's own information and the surgeon's interpretation of radiographic images [1]. Gait analysis can be used to assess knee function before and after knee arthroplasty. It provides a novel method for objectifying knee function [3-5]. The “gold standard” for gait analysis is advanced 3-D motion capture analysis technology [6], but these systems are costly and require a specialized laboratory. Inertial measurement units (IMUs) have been proven to be valid and in high agreement with motion capture technology while being cheap, mobile, and easy to use [7]. IMUs are increasingly realistic to use in a clinical setting. A multicenter randomized double-blinded controlled trial of 350 patients aimed at investigating the patient-reported and clinical differences between unicompartmental knee arthroplasty (UKA) and total knee arthroplasty (TKA) is ongoing at our institution [8]. This exploratory study was initiated to explore the sagittal knee gait changes of these patients. The specific study aim was to investigate the developments of sagittal knee gait of randomized and blinded patients, receiving either UKA or TKA. We hypothesized that the patients achieve greater walking speeds, greater range of motion, and faster and more powerful knee swings, measured in the sagittal plane. Gait analysis has the potential to offer new and meaningful data to surgeons when assessing the postoperative result following knee arthroplasty, which is the future perspective of this study.

## Materials and methods

**Figure 1:**
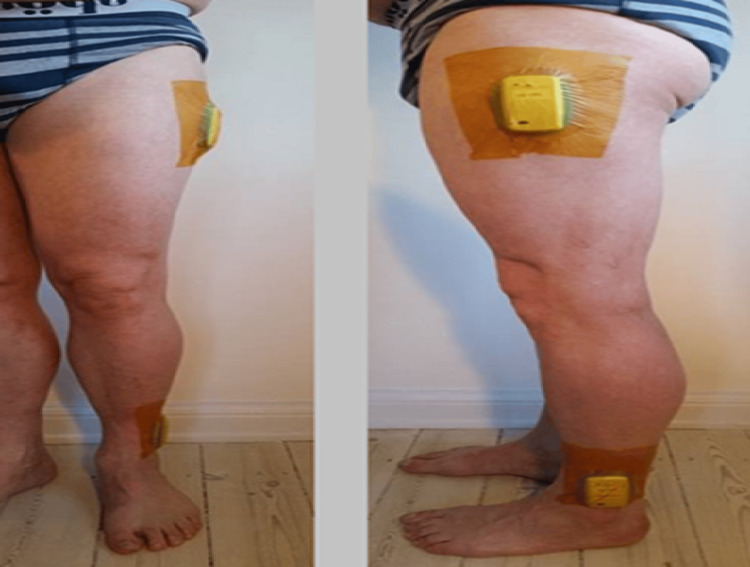
Placement of inertial measurement units (IMUs) IMUs are placed immediately below the greater trochanter laterally and immediately above the lateral malleolus. The picture is published with the consent of the participant.

This study was an exploratory study of the developments of sagittal knee gait following knee arthroplasty. Patients in this study were included prospectively from March 2018 to October 2020, The patients participated in an ongoing national multicenter double-blinded randomized controlled trial (RCT) [8]. The RCT is registered at clinicalTrials.gov (registration no. NCT03396640) and complies with the Consolidated Standards of Reporting Trials (CONSORT) guidelines [9,10]. All patients gave their written and informated consent to both studies. The Danish Ethics Committee approved the initiation and completion of the studies (approval no. H-16037372). The Strengthening of the Reporting of Observational Studies (STROBE) guidelines were applied to this study. Only patients with radiographically and perioperative confirmed anteromedial-osteoarthritis were included. Patients were randomized during the surgery to receive either cementless medial Oxford partial knee phase 3-alpha (UKA) or cemented TKA. The type of TKA used was the surgeons’ preference. Regardless of the implant received, the same surgical approach was applied (midline incision). Measurements of gait were made two weeks before the surgery, four months after surgery, and again one year postoperatively. Patients and the research group were blinded for implant type one year postoperatively. Only patients included at one of the participating hospitals were offered inclusion in this study. The patients were equipped with two IMUs (ISENS-100, Icura Aps, Copenhagen, Denmark) connected to a smartphone. One sensor was positioned at the lateral aspect of the proximal thigh and the other distally at the lower leg (Figure 1).

**Figure 2:**
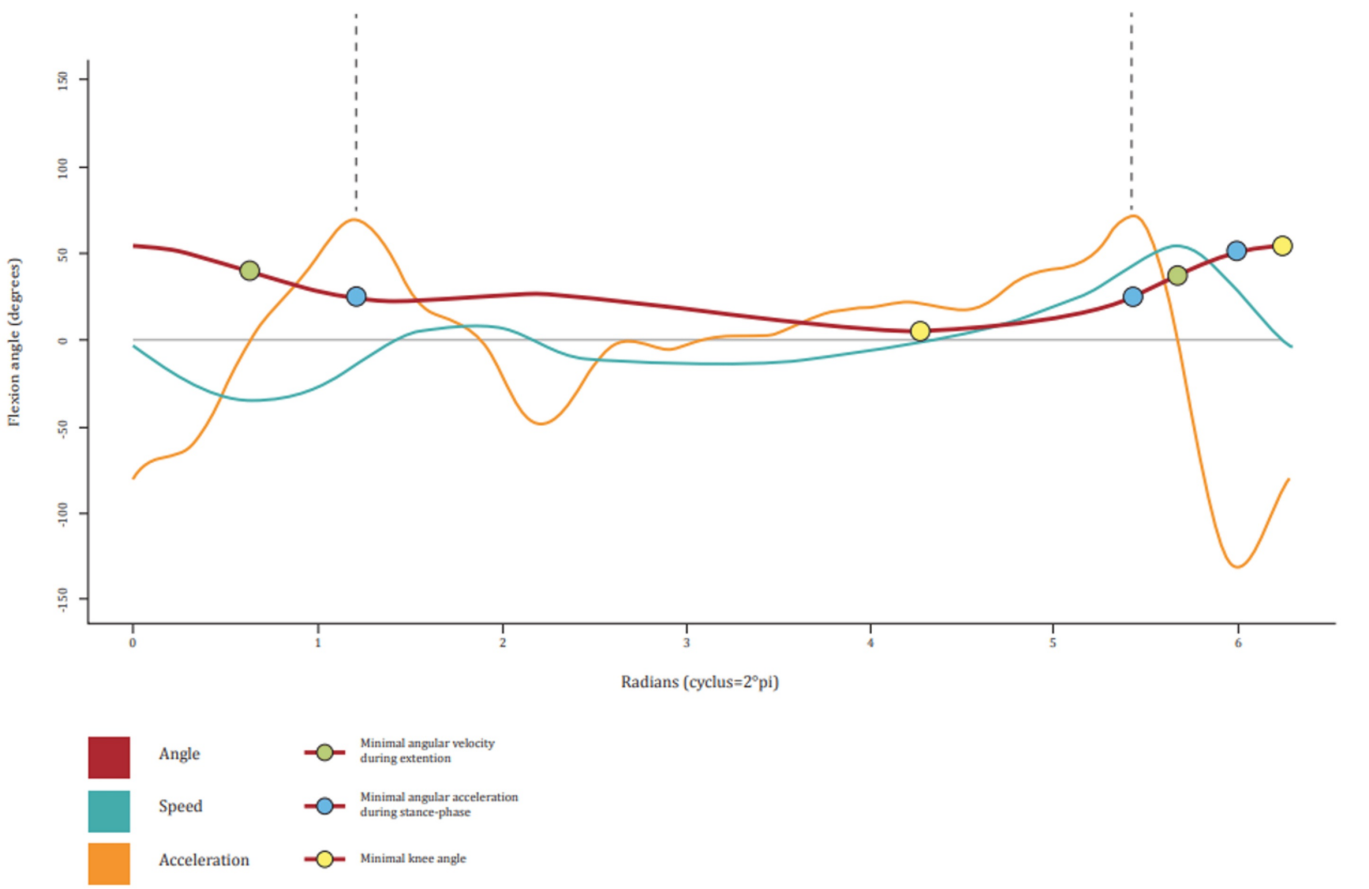
Gait cycle with measured sagittal knee angles, angular velocities, and angular accelerations

The IMUs were calibrated automatically and subsequently manually before measurements (using a goniometer), and recording was controlled by a custom-made app. The sagittal knee joint angle was measured with a nominal frequency of 20 Hz. Each sensor weighed 27 grams and its dimensions were 68x42x15 millimeters. The sensors consisted of an accelerometer, gyroscope, and magnetometer. Patients were first asked to walk for a six-minute familiarization period on the treadmill at level walking [11]. Patients were then asked to adjust the speed of the treadmill to their maximal walking speed, at which recording for data collection began. The duration of the recording used for analysis was 60 seconds. Our algorithm used event landmarking for identifying the sagittal knee swings in our data [12]. The Lomb-Scargle algorithm was used to determine the stride frequency. Within each period, the maximal flexion was landmarked, and a Fourier series was used to fit the average gait cycle from raw data [13]. The Fourier series allowed easy calculation of first and second derivatives and integrals, which in turn allowed the calculation of angular velocity and acceleration (Figure 2).

Nine different gait parameters were calculated from the Fourier expansion. We focused on spatiotemporal parameters (stride frequency, walking speed), range of motion, the average knee angle during a stride, angular velocity (maximum flexion and extension velocities), and angular accelerations (maximum flexion and extension acceleration during the swing phase and maximum flexion acceleration after heel strike) (Table 1). 

**Table 1:** An overview of calculated parameters from the average gait cycle.

Type of data	Parameter	Unit of measurement and description
Spatiotemporal data	Stride frequency	Strides per second; A stride is defined as the point of maximal flexion in a swing, to the following point of maximal flexion in the next swing (s^-1^).
	Walking speed	Meters per second
Angular data	Range of motion	Difference between maximal and minimal angle measured (in degrees). The amplitude corresponds to the angular range from maximal extension to maximal flexion. It is interpreted as the range of motion (ROM).
	Average knee angle	The area under the average gait cycle curve is divided by measurement duration. It is a measure of the average knee angle during the gait cycle.
Velocity data (first derivative)	Maximal extension velocity during swing	The smallest value of angle velocity during the gait cycle. (angles/second). The highest angular speed of an extension movement.
	Maximal flexion velocity during swing	The largest value of angle velocity during the gait cycle. (angles/second). The highest angular speed of a flexion movement.
Acceleration data (second derivative)	Maximal flexion acceleration during a swing	The maximal acceleration measured during flexion in swing-phase (angles/second^2^).
	Maximal extension acceleration during swing	The maximal acceleration measured during extension in swing-phase (angles/second^2^).
	Maximal acceleration after heel-strike	The maximal acceleration measured after the heel strike during stance phase (angles/second^2^).

Comparisons over time were done using a mixed effects model with a random subject-specific effect to account for the correlation within subjects. A comparison of UKA and TKA patients were not performed due to the low sample size. P-values below 0.05 were considered significant. The models were validated considering the goodness of fit plots of the residuals, and in some cases, the models were fitted with and without outliers to check that the results were robust. The normal distribution of parameters was investigated using exploratory statistics such as histograms and probability plots. Model summaries were presented in a table. We focused on regression coefficients because the focus of this study was to investigate the changes in sagittal knee gait over the observation period. All analyses were performed using R Statistical Software (v4.1.2; R Core Team 2021, R Foundation for Statistical Computing, Vienna, Austria, https://www.r-project.org/). We did not perform a power analysis before the initiation of this study because of its exploratory nature. This article was previously posted to the Research Square preprint server on January 29, 2024.

## Results

**Figure 3:**
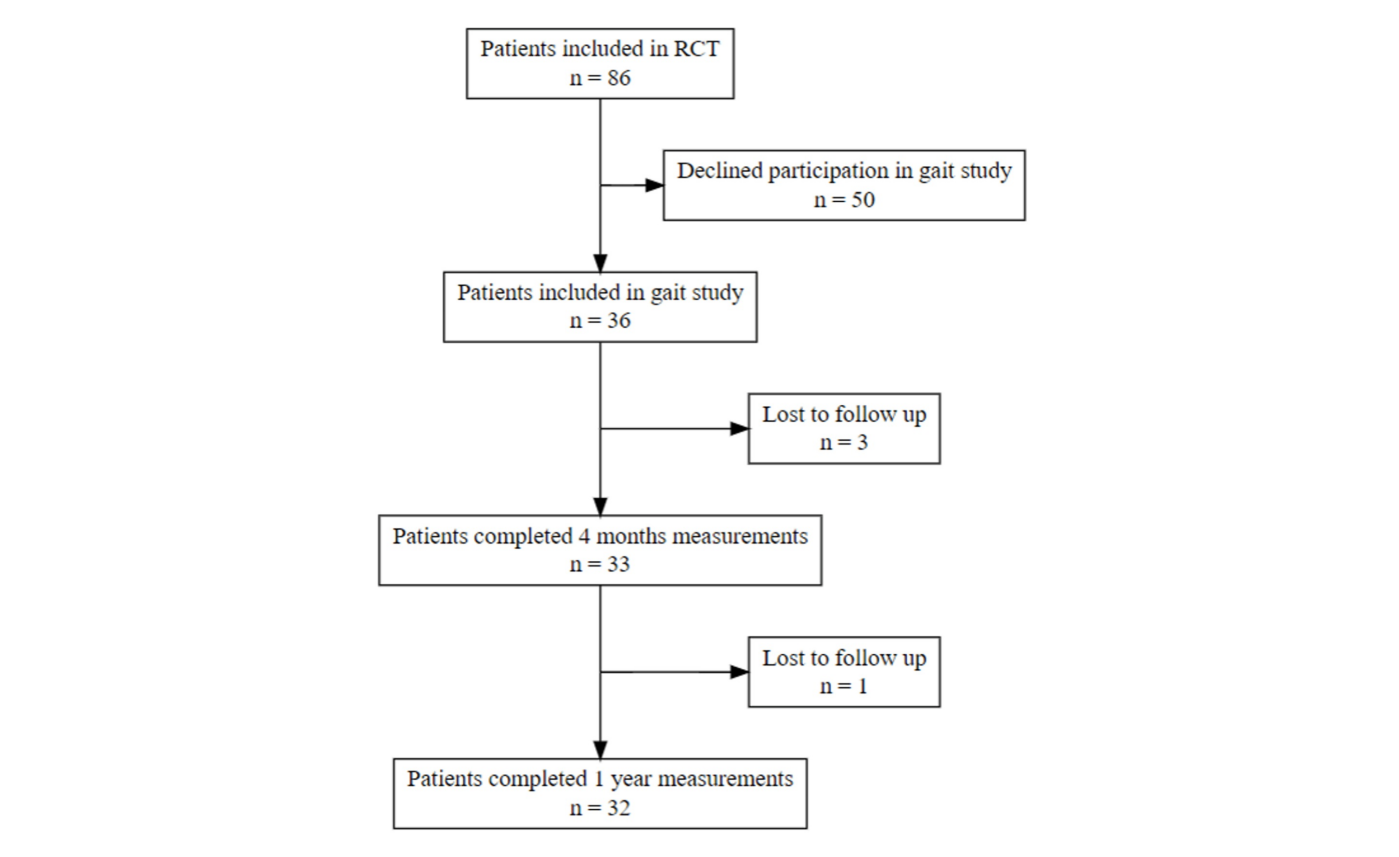
Flowchart depicting the process of inclusion, measurements, and loss to follow-up.

Exploratory statistics revealed that the 10 outcome parameters were normally distributed. The number of patients included at our institution into the RCT was 86. Of these 86 patients, 36 patients (21 UKA and 15 TKA) consented to be included in the gait study and they all completed preoperative measurement. Following surgery, 33 of the 36 patients completed four-month measurements and 32 completed one-year measurements (Figure 3).

No considerable differences in patient demographics were found between the patients participating in the gait analysis when grouped by implant type. Also, we did not find any considerable differences between patients participating in the gait analysis, the patients who declined participation in the gait measurements, and the population of patients participating in the RCT. Therefore, we considered the groups to be comparable (Table 2).

**Table 2:** Demographics of the participanting patients in gait analysis, patients who declined participation, and all patients participating in the randomized trial (RCT). UKA: unicompartmental knee arthroplasty; TKA: total knee arthroplasty; RCT: randomized controlled trial

	UKA (n = 21)	TKA (n = 15)	Declined participation in gait study (n = 50)	All patients participating in RCT (n = 350)
Sex (male:female)	15:6	8:7	26:24	185:165
Age (years)	68.1±5.7	68.8±5.7	63.76±8.5	65.1 ± 7.4
BMI (kg/m^2^)	28.6±4.4	29.2±3.6	28.87±4.3	29.2±4.4
Height (m)	1.7±0.11	1.7±0.1	1.8±0.1	1.7±0.1
Passive extension (degrees)	124±8	124±12	123±12	124±12
Passive flexion (degrees)	1±3	3±4	2±3	1±3
The values are indicated as means with standard deviation. For intergroup comparison, Student’s t-test was used for age, BMI, and height.				

Stride frequency increased by 0.1 (p = 0.01) and 0.2 (p<0.001) at four months and one year postoperatively. Walking speed increased by 0.2 m/s (p<0.001) and by 0.3 m/s (p<0.001) after four months and one year. The sagittal range of motion of the gait cycle increased by 7 degrees (p<0.001) after one year. The average knee angle during walking increased by 2 degrees (p =0.048) and by 4 degrees (p<0.001) after four months and one year. The maximal sagittal extension velocity during the swing increased significantly by 72 degrees/second (p<0.001) after one year. The maximal sagittal flexion velocity during the swing increased significantly by 49 degrees/second (p = 0.006) after one year. The maximal sagittal flexion acceleration during the swing increased by 565 degrees/second^2^ after one year (p = 0.02). The maximal sagittal extension acceleration during the swing increased by 1168 degrees/second^2^ (p = 0.03) after one year. The maximal sagittal flexion acceleration after heel-strike increased significantly by 1549 degrees/second^2^ (p <0.001) after one year (Table 3).

**Table 3:** Mixed effects model. Regression coefficients with corresponding confidence intervals and p-values are listed for each time and parameter. The p-values represent comparison of all patients (TKA and UKA patients) at the time of measurement with the preoperative measurement as reference. P-values below 0.05 were considered significant.

		Stride Frequency (s^-1^)	Walking speed (meters/second)	Range of motion (degrees)	Average knee angle (degrees)	Maximal extension velocity during swing (degrees/second)	Maximal flexion velocity during swing (degrees/second)	Maximal flexion acceleration during a swing (degrees/second^2^)	Maximal extension acceleration during swing (degrees/second^2^)	Maximal acceleration after heel-strike (degrees/second^2^)
4 months	Estimate	0.1	0.2	2	2	22	16	122	395	610
	95 % confidence interval	0.02-0.1	0.2-0.3	-1 to 6	0.06-4	-5 to 49	-8 to 41	-390 to 638	-252 to 1,046	-218 to 1,444
	p-value	p = 0.01	p <0.001	p = 0.2	p = 0.048	p = 0.1	p = 0.2	p = 0.6	p = 0.24	p = 0.2
1 year	Estimate	0.2	0.3	7	4	72	49	565	1168	1549
	95 % confidence interval	0.1-0.2	0.3-0.4	4-10	2-6	44-100	25-74	113-1159	589-1902	705-2398
	p-value	p <0.001	p < 0.001	p <0.001	p <0.001	p <0.001	p = 0.006	p = 0.02	p = 0.03	p <0.001

## Discussion

This study aimed to investigate pre- to postoperative changes in sagittal knee gait after UKA and TKA nested in a blinded RCT. We believe this to be the first study to investigate sagittal knee angle acceleration using IMUs in a blinded and randomized population. In our study population, it was found that the patients developed faster walking speed with greater stride frequency, greater range of motion during walking, and faster and more powerful knee swings. Previous studies have found that as gait improves postoperatively, so do the patient-reported outcome measures [14]. A study found a correlation between increasing Western Ontario and McMaster Universities Arthritis Index (WOMAC) scores and increasing values of walking speed and cadence, meaning that improvements in gait may be a clinically valid measure of knee status [15]. Another study found that angular velocity increases dramatically following knee arthroplasty, which agrees with our findings [16]. However, as previous studies are heterogeneous in their design, the number of participants, the time at which patients are measured postoperatively, method of data acquisition and analysis, it is difficult to compare the results directly. Angular velocity and acceleration may be more informative expressions of improved knee kinematics following arthroplasty because patients with pain or instability of the knee are likely to reduce the amount of force exerted on their knee joint. This is in concordance with the hypothesis of other studies [17]. If the amount of force that is exerted on the knee joint is increased, the patient will likely have a greater sensation of pain. Angular velocity and acceleration may thus be the most sensitive parameters for assessing the sagittal knee gait function. However, the study by Calliess et al. found that walking on a level surface at a normal speed correlates less strongly with clinical outcome outscores [17]. This fact is especially true for healthy young adults with normal gait. We theorize that pathologies in gait may be more clearly expressed in patients walking at their self-chosen maximal speed. Using the second and third derivatives of the measured sagittal knee angles, as we did, will be sensitive to even small changes in gait, so small increases in angular velocities will produce large increases in angular accelerations. For this reason, we believe that angular velocities will be sensitive outcome parameters in the gait analysis of knee arthroplasty patients [17]. 

No statistical comparison was made in this study due to the low sample size, making comparisons very insecure. In a systematic review, Nha et al. found no differences between the gait of the two groups except shorter stride length in the TKA group compared with the UKA group [18]. Our results are in agreement with this finding, as no obvious differences between the groups are discernible, although we did not test this. In general, studies comparing UKA and TKA have found similar clinical outcomes for the first one to two years [19-21]. Wiik et al. found that downhill walking gait may differ between UKA and TKA, as they found that patients with UKA walked faster than TKA patients when walking downhill. They hypothesized that this finding may be due to an intact anterior cruciate ligament in the UKA group (which is retained during surgery) [22]. The anterior cruciate ligament (ACL) is important for the proprioception of the knee, and its removal during the insertion of TKA could be a confounding factor for differences in gait between UKA and TKA, which would be interesting to investigate in future studies. There were limitations to our study. The magnetometer in the sensor was vulnerable to magnetic interference, but steps were taken to ensure that no large metallic objects were adjacent to the sensors. A common limitation when using IMUs is measurement errors due to possible loosening of the sensors. An adhesive surgical tape was used to prevent these errors, but wobbling can still occur in patients with excess soft tissue on the hip [23]. No adverse events following the application of the tape were reported from our patients. Another limitation was the low number of participants, which makes our study vulnerable to selection bias. However, we found no signs of disparity in age, height, BMI, or gender between the study population and the RCT population. As a result, we consider our sample population to be representative. In addition, the number of participants was regarded as sufficient in an exploratory study of this kind. Precise quantifications of forces were not possible with our design, as we used IMUs and not force plates. However, we focused on the measurement of differences over time. 

## Conclusions

In conclusion, we have reported pre- to postoperative changes in sagittal knee gait following knee arthroplasty. Parameters such as walking speed, range of motion, sagittal knee angle velocity, and acceleration increased significantly following the two types of knee joint arthroplasty. These increases are indicative of improved sagittal knee gait patterns. We found that UKA and TKA seem to develop equal changes in sagittal knee gait following surgery. Angular acceleration may be the most indicative parameter for assessing the sagittal knee gait function.
